# Early Sexual Debut and the Associated Factors among School Going Adolescents in Selected Schools in Kenya

**DOI:** 10.24248/eahrj.v7i2.734

**Published:** 2023-11-30

**Authors:** Gideon Mauti Ogutu, Scholastica Muthoni Chege

**Affiliations:** a Department of Community Health and Development, The Catholic University of Eastern Africa, Nairobi, Kenya

## Abstract

**Background:**

Early sexual debut among adolescents' results in sexual and reproductive health consequences including unplanned pregnancies, pregnancy complications and sexually transmitted diseases.

**Objective:**

This study investigated the factors influencing early sexual debut among 13–19 years old students in secondary schools in Kiambu County, Kenya.

**Methodology:**

A descriptive cross-sectional study was carried out in two secondary schools in Kiambu County. A sample of 208 was calculated using Fischer's two stage formula and proportionately distributed per school population size. Students in each school were stratified into classes and respondents were selected by simple random sampling from each stratum. Responses were sought using self-administered questionnaires.

**Results:**

Among the respondents 11(66.5%), 48(28.7%) and 8(4.8%) were aged 13–15, below 13 and above 15 years, respectively. Eighty-four (50.2%) respondents were male, 105(62.9%) lived with both parents while 41(24.6%) lived with mothers. Early sexual debut was (60.5%), being higher in males, 57 (67.9 %) compared to females, 44 (39.6 %). More than half of the respondents, 117(69.6%), thought sex before marriage was appropriate, among whom 75(64.1%), have had sex. Most of those who reported sexual debut, 69.9%, said that sexual encounter happened during school breaks. Reasons for refraining included fear of guardian (49.4%) and fear of HIV/AIDS 89(52.7%). However, more than two thirds, 71(70.3%) of those who feared HIV/AIDS and STI as a consequence of early sexual debut have had sex. Gender (P=.032), knowledge on sex (P=.025), use of mobile phones (P=.019), peer pressure (P=.046) and poverty (P =.037) were significantly associated with early sexual debut.

**Conclusion:**

A significant proportion of secondary school adolescents were engaged in early sexual debut. Thus, public health interventions should consider the broader determinants of early sexual debut, including the ecological factors in which the behavior occurs.

## INTRODUCTION

Early sexual debut is sexual activity before the age of 18 years.^1^ Adolescents, initiate early sexual relationships exposing them to the risks of unintended pregnancies, and contracting sexually transmitted diseases among others. ^2^ In Kenya, there is a high prevalence of early pregnancy^3^ with more teenagers from poorer households, 26%, beginning childbearing compared to 10% from wealthier households.^4^

Early sexual debut, especially among women leads to longer period of exposure to the risk of acquiring Human Immunodeficiency Virus (HIV) and predispose them to engage in risky sexual behavior with increased exposure to sexually transmitted infections (STI's), pelvic inflammatory disease (PID) and HIV. ^5^ High incidence of teenage pregnancy limits education and economic opportunities for the youth hence increasing poverty. ^6^

Age at sexual debut varies from place to place and among different individuals, ranging from a mean age of 11 years to 17 years.^7,8^ Age as a result of physical development has a dramatic effect on adolescents' sexual behavior.^9^ Estimates of Antenatal Care (ANC) coverage at lower decision-making units (sub-county^3,2^ On the other hand, most adolescents have sex for the first time because of social pressures, socioeconomic and environmental conditions. ^10,11^ Additionally, low educational level of parents and poverty contribute to early sexual debut.^12,13,14^ The internet, content in mass media and peer pressure are important predictors of early sexual debut.^13–15^

Pressure on females is thought to rise from their subordinate position to males^15^ while males are pressured to be highly sexually attractive in order to be socially recognized as physically mature.^16,13^ Girls generally report earlier sexual debut and with older partners.^17,18^ Schooling is protective against early sexual debut since sexual debut is positively associated with having permanently dropped out of school and having never attended school.^13^

### Significance of the Study

Youths undergo physiological changes that could drive them to engage in risky sexual behaviors. Understanding sexual behaviors among adolescents would help in attainment of safe sex practices hence reduce the cases of adolescent pregnancy, STI and HIV infection, thereby promoting adolescents to achieve their educational goals. The study findings add knowledge for implementers and policy makers in using effective strategies for behavior change promotion among the adolescents. The study was also expected to provide information to health care providers that could be used in improving the already existing adolescent friendly reproductive health programs.

Therefore, this study sought to identify factors associated with early sexual debut among adolescent (13–19 years old).

## METHODOLOGY

### Study Setting, Design, and Sampling Study Design.

A cross-sectional, descriptive study design with quantitative data collection techniques was used.

### Study Setting and Site

Two secondary schools were conveniently sampled in Kiambu County, near Nairobi County in Kenya. The secondary schools were of both male and female genders.

### Sample Size Determination and Sampling Criteria

Fischer's two stage formula was used to determine the sample size. The total population of the students was 453. Using the formula, where, n=the desired sample size, Z=the standard normal deviation at the required confidence level (1.960 with the confidence level of 95%), D=the level of statistical significance set (0.05), and P= proportion in target population (50%=0.5), a sample size of 384 was calculated. The actual sample size was further adjusted using the formula; where, nf = desired sample size and N=the estimate of population size (453 students) to give 208 students. The numbers were proportionately distributed with 68 students in Riabai school and 137 students in Kiambu Township school. Each school was then stratified into four levels: form one being the lowest class while form four was the highest. Sample size in each school was finally proportionately distributed per class size. Individual respondents were selected by simple random sampling using a sample frame of all students present in the class.

### Inclusion and Exclusion

All students who consented and who were present at the time of data collection were included. Any student who was absent was excluded.

### Administration of Questionnaires

A self-administered questionnaire was filled by each respondent. Each sampled student was given adequate privacy ensuring no other student or research assistant was close enough to see responses as were written. This ensured research participant confidence in privacy of responses. Data was collected on sexual debut and related characteristics including demographic characteristics, individual factors, socio-economic factors and perceived consequences of early sexual debut.

A total of 208 questionnaires were administered. However, 167 questionnaires were returned representing 80.2% response rate. Of the 31 that were considered non response, 17 were returned without any response while 14 only had responses on socio-demographic characteristics and were considered non response.

### Data Quality

Each filled questionnaire was sealed in a non-labeled envelop for anonymity before being handed over to the research assistant. The research assistant collected all questionnaires and handed over to the research administrator who confirmed collection of all. The custodian of all answered questionnaires was the principal investigator and no other individual. The principal investigator was responsible for enrollment of the students to the study assisted by the research assistants. A one-day training was conducted where the participants were informed about the objectives, benefits and risks of the study. The questionnaires were administered with codes used for each of them.

### Data Analysis

Data was coded and entered on excel by the principal investigator. Cleaning was done and completeness checked. Data was exported to Statistical Package for Social Science (SPSS) version 21 (SPSS, Inc., Chicago I). Categorical variables were analyzed descriptively using frequency and percentages. Chi-square test was used to find statistical associations between independent and dependent variables. Associations were established using Chi square at a 95% confidence interval. A *P* value of ≤ was declared statistically significant.

### Ethical Considerations

Informed consent was obtained from each student 18 years of age and above. Assent for students under 18 years was obtained from the students by the school principal, who received delegated responsibility over the students after informing the specific parents. Privacy was ensured for each respondent by giving adequate space of at least 3 meters to the next respondent. Each questionnaire was anonymized by use of a code and no names were revealed. The study protocol received ethical review and clearance by the Kenyatta National Hospital-University of Nairobi Ethics Review Committee.

## RESULTS

### Sociodemographic Characteristics of Respondents

About half of the students, 84(50.3%) were male while 83(49.7%) were female. More than two thirds of respondents, 111(66.5%) were aged between 13 to 15 years. About two thirds of respondents, 105(62.9%) lived with both parents while almost a quarter, 41(24.6%) were living with their mothers as shown in Table 1.

**TABLE 1:** Sociodemographic Characteristics of Respondents

Demographic characteristics	(n)	(%)
Gender		
Female	83	49.7
Male	84	50.2
Students age		
Below 13	48	28.7
13–15	111	66.5
Above 15 years	8	4.8
Students class		
One	46	27.6
Two	45	26.9
Three	45	26.9
Four	31	18.6
Students' Guardian		
Father	11	6.6
Mother	41	24.6
Both	105	62.9
Other	10	5.9

### Prevalence of Sexual Debut and the Related Individual Factors

Early sexual debut was 101(60.48%), highest among males, 57(67.9 %) as compared to females, 44(39.6 %). More than half of the adolescents who reported early sexual debut, 27(55.1%) had their sexual debut in the age group 13 to 15 years followed by those aged below 13 years, 4(36.4%). Two thirds of sexually active girls, 22(61.1%), reported having sexual debut with older partners as compared with more than two thirds, 28(68.3%) of sexually active boys who had sexual debut with age mates, or with younger partners.

Early sexual debut was higher among those who believed sex before marriage was appropriate, 75(64.1%); and among adolescents who had enough knowledge on sex, 38(73.1%). Socialization also seems to influence early sexual debut since about two thirds, 60(62.1%), of those who easily mingled with others, reported early sexual debut. It was also noted that among those who experienced early sexual debut, it occurred more during school breaks, 40, (65.6%). Age difference between sexual partners (*P*=.032) and gender (*P*=.05) were individual factors significantly associated with early sexual debut, Table 2.

**TABLE 2:** Sexual Debut and the Related Individual Factors

Factor	Has had sex (engaged)		Totals	P Value
	Yes	No		
Female	44(53.0%)	39(47.0%)	83(100.0%)	.05
Male	57(67.9%)	27(32.1%)	84(100.0%)	
Total	101(60.48%)	66(39.52%)	167(100.0%)	
Age at sexual debut				
Below 13	4(36.4%)	7(63.6%)	11(100.0%)	.214
13–15	27(55.1%)	22(44.9%)	49(100.0%)	
16–18	15(34.1%)	29(65.9%)	44(100.0%)	
Above 18	1(50.0%)	1(50.0%)	2(100.0%)	
Age difference with sexual partner at sexual debut				
	Females	Males		
Same age	13(31.7%)	28(68.3%)	41(100.0%)	.032
Younger	12(41.4%)	17(58.6%)	29(100.0%)	
Older	22(61.1%)	14(38.9%)	36(100.0%)	
**Has had sex (engaged)**				
		Yes	No	
Happy with how I look	Yes	42(42.0%)	58(58.0%)	.472
	No	31(47.7%)	34(52.3%)	
Sex before marriage	Appropriate	75(64.1%)	42(35.9%)	.143
	Inappropriate	26(52.0%)	24(48.0%)	
Enough knowledge	Yes	38(73.1%)	14(26.9%)	.025
	No	63(54.8%)	52(45.2%)	
Easily mingle with opposite sex	Yes	60(62.1%)	41(37.9%)	.726
	No	41(59.4%)	25(40.6%)	
Easily maxe menus	Yes	47(57.4%)	26(42.5%)	.363
	No	54(64.4%)	40(35.6%)	
School breaks	Yes	40(65.6%)	21(34.4%)	.305
	No	61(57.5%)	45(42.5%)	

### Socioeconomic Factors Contributing to Early Sexual Debut

Respondents believed that media sources, (81.5%), social belonging (78.7%), peer pressure (76.2%) and drugs and alcohol use (67.6%), influenced early sexual debut among adolescents. However, two thirds, (60.0%), believed poverty would influence sexual debut among adolescents, (Table 3).

**TABLE 3:** Sociodemographic Factors Influencing Sexual Debut among Adolescents

Living arrangement	Early sexual debut			X2	P value
	Yes n (%)	No n (%)	Total n (%)		
Father	6(5.9)	5(7.6)	11(6.59)	0.741	.864
Mother	23(22.8)	18(27.3)	41(24.55)		
Both	66(65.3)	39(59.1)	105(62.87)		
Other	6(5.9)	4(6.1)	10(5.98)		
Ever had discussion on sex with parent					
Yes	45(60.0)	30(40.0)	75(44.91)	0.013	.909
No	56(60.9)	36(39.1)	92(55.09)		
Sibling influence					
Yes	22(21.8)	13(19.7)	35(20.96)	0.105	.746
No	79(78.2)	53(80.3)	132(79.04)		
Mobile phones influence sexual debut					
Yes	82(70.1)	48(28.8)	130 (77.84)	9.935	.019
No	19(11.4)	18(10.9)	37 (22.16)		
Peer pressure					
Yes	84(63.3)	62(37.2)	146 (87.43)	8.192	.046
No	17(10.29)	4(2.4)	21 (12.57)		
Drugs and substance abuse					
Yes	80(59.1)	49(29.4)	129 (77.25)	4.318	.229
No	21(12.69)	17(10.2)	38 (22.75)		
Poverty					
Yes	66(65.3)	53(44.5)	119 (71.26)	4.360	.037
No	35(34.7)	13(19.7)	48 (28.74)		
Internet					
Yes	61(61.6)	38(38.4)	99 (59.28)	0.132	.717
No	40(58.8)	28(41.2)	68 (40.72)		

Despite two thirds of the adolescents, 105(62.87%), living with both parents, more than two thirds among them, 66 (65.3%), still reported early sexual debut. More than half of respondents, 92(55.09%), had never had any discussions about sex with their parents or guardian. Still, about 22(59.5%), of those who had discussions about sex with guardians reported sexual debut. About 130(77.84%) among all respondents said mobile phones influenced sexual debut, among whom, 82(70.1%), reported sexual debut, Table 3.

About two thirds, 84(63.3), among the 146(87.43%) who said early sexual debut resulted from peer pressure had engaged in sex. Majority of adolescents, 129(77.25%), reasoned that drug and substance abuse led to early sexual debut among youths. Of these, more than half, 80(59.1%), reported having engaged in sexual activities. Further, 119(71.26%) of all respondents, believed that poverty could force them into early sexual debut. More than two thirds, 66(65.3%), among those who thought poverty resulted in early sexual debut reported having engaged in sex. Media, social belonging, peer pressure, drugs and substance abuse were perceived to greatly influence early sexual debutFigure 1.

**FIGURE 1:**
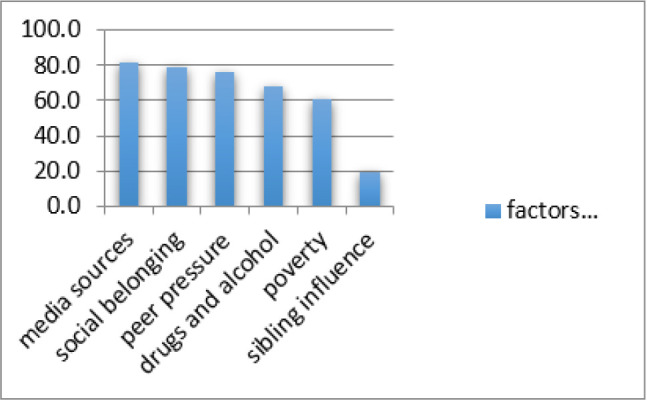
Factors Influencing Early Sexual Debut

Individual level factors such as level of sexual knowledge, (*P*=.025), peer pressure (*P*=.046), use of mobile phones (*P*=.019), and poverty (*P*=.037) were significantly associated with early sexual debut Table 3.

### Reasons for Refraining From Early Sexual Debut

Among those who refrained from sex, more males, 48(57.1%) compared to females, 41(49.4%), refrained from sex because of fear of guardian. Still among those who refrained, contrary to perceptive beliefs, more males, 46(54.8%), compared to females 43(51.8%) refrained due to fear of pregnancy. The risk of HIV infection doesn't appear to inhibit sexual debut since only slightly more than half males, 47(56.0%) and 42(50.6%) of female adolescents refrained from sex because of fear of HIV/AIDS. Again, 48(57.8%) of females and 49(57.0%) males didn't think they were too young to engage in sex. Additionally, 44(52.4%) of males and 46(55.4%) females considered refraining until after completion of studies. It was also noted that religious beliefs may not be a refraining factor since 52(61.9%) and 49(59.0%) among males and females, respectively, did not consider engaging in sex to be against their religious beliefs. Table 4.

**TABLE 4:** Refraining Factors and Gender of Respondents

Factor	Female N, (%)	Male N, (%)
Fear of guardian		
Yes	41(49.4%)	48(57.1%)
No	42(50.6%)	36(42.9%)
Risk of pregnancy		
Yes	43(51.8%)	46(54.8%)
No	40(48.2%)	38(45.2%)
Waiting for the right partner		
Yes	38(45.8%)	41(48.8%)
No	45(54.2%)	43(51.2%)
Risk of HIV/AIDS		
Yes	42(50.6%)	47(56.0%)
No	41(49.4%)	37(44.0%)
I am too young		
Yes	35(42.2%)	37(43.0%)
No	48(57.8%)	49(57.0%)
Waiting to complete studies		
Yes	37(44.6%)	44(52.4%)
No	46(55.4%)	40(47.6%)
Against religious beliefs		
Yes	34(41.0%)	32(38.1%)
No	49(59.0%)	52(61.9%)

### Perceived Consequences of Early Sexual Debut

Although 120 (71.4%) of respondents perceived that early sexual debut would result in HIV/AIDS and STI, more than two thirds, 71(70.3%) of them have had sex. Almost 74(44.0%) of respondents believed it resulted in heartbreak and depression. Despite most females, 67(80.7%) and more than half of males, 56(66.7%) believing it would result in school dropout, 71(57.7%) reported early sexual debut.

More males, 52(62.7%), perceived pregnancy to be a consequence of early sexual debut compared to 31(37.3%) females. Early sexual debut was higher, 56(64.4%) among those who didn't fear pregnancy 45(56.3%) compared to those who feared pregnancy, 45(56.3%), Table 5.

**TABLE 5:** Perceived Consequences by Gender

consequence	Gender			Had sex	
	Female n=83	Male n=84	P value	Yes 101(60.5%)	No 66(39.5%)
HIV/STI					
Agree	59(49.2%)	61(50.8%)	0.825	71(70.3%)	49(40.8%)
disagree	24(28.9%)	23(27.4%)	(0.049)	30(59.2%)	17(25.8%)
Heartbreak and Depression					
Agree	42(56.8%)	32(43.2%)	2.647	42(41.6%)	32(48.5%)
disagree	41(44.19%)	52(61.9%)	(0.120)	59(58.4%)	34(51.5%)
School dropout					
Agree	67(80.7%)	56(66.7%)	4.451	71(57.7%)	52(42.3%)
disagree	16(19.3%)	28(33.3%)	(0.039)	30(29.7%)	14(21.2%)
Pregnancy					
Agree	31(37.3%)	49(58.3%)	7.366	45(56.3%)	35(43.8%)
disagree	52(62.7%)	35(41.7%)	(0.008)	56(64.4%)	31(35.6%)
Poor relationship with parent					
Agree	35(43.2%)	46(56.8%)	2.651	49(60.5%)	32(39.5%)
disagree	48(55.8%)	38(44.2%)	(0.104)	52(50.5%)	34(39.5)

## DISCUSSION

About two in every three adolescent students reported early sexual debut, representing a prevalence of 60.48%). Of these, 67.9% were males while 53.0% were females. The high prevalence of early sexual debut among the adolescent students is of great concern especially regarding its association with sexually transmitted diseases. Elsewhere, early sexual debut was found to be associated with factors that may increase a young person's risk for HIV infection.^19^ These findings are higher than the national prevalence.^3^ This finding was higher than the finding from Oslo,^20,1^ but slightly compares with a study in Humera, Ethiopia, which reported a 63.3% prevalence.^21^

Early sexual debut was associated with gender since females were 47.0% less likely to report sexual debut compared to males. This finding is slightly lower than that in another study.^22^ however, about the sexual values, perceptions and subsequent sexual practices of youth whose sexual debut occurs while using alcohol/drugs. Methods. A cross-sectional anonymous online survey was conducted in April-August 2012 among undergraduate and graduate university students (aged 18 to 30 The gender prevalence was slightly higher than the national prevalence reported from the KDHS 2008–2009 survey.^23^ The possible explanation is that males are pressured to be highly sexually attractive in order to be highly socially recognized as physically mature. ^16^ Although in this study, age at menarche was not investigated, other findings concluded that women with early menarche start sex and marry early.^24^

Girls seemed to have sexual debut with older partners compared to male students who either had it with age mates or younger partners. The age difference between sexual partners was significantly associated with early sexual debut, (*P*=.032). These findings are similar to those in a study in Norway.^17^ and compares to others in a study in South Africa where 71.5% of females compared to 17.6% males had sex with older partners.^19^ The possible explanation is that when girls mature physically at an early age, they begin menarche early and appear older than their age, they are also more likely to initiate sex early with older partners. ^25^ Additionally, girls experience more pressure on due to their subordinate position to males.^15^

Sexual debut was higher among those who believed sex before marriage was appropriate.

In this study, early sexual debut was higher among adolescents who had adequate knowledge on sex. With increased access to internet and mobile phones, adolescents acquire extensive information related to sexuality, which may be misguiding and can have a significant negative impact on the sexual behaviors.^26^

Sexual debut was higher among students who easily mingled with others. These findings concurs with other findings elsewhere which found that high social self-perception is positively associated with early debut. ^20^

The study showed that parental role in the adolescents' sexual life is critical. Despite 62.87% of the students living with both parents, 65.3% of them reported early sexual debut. Further, more than half had never had any discussions about sex with their parents or guardian with 59.5% among them having had early sexual debut. School holidays when adolescents are at home interacting with their peers is a time of social experimentation, especially with poor parental supervision and connectedness. The lack of parental engagement has been described to be associated with early sexual debut in African countries. ^27^

Most students were in agreement that media sources of explicit sex content influenced early sexual debut. Access to smart phones and internet has increased in Kenya, and most youths increasingly interact with uncensored internet content. This suggestions by the participants agree with another which explained the impact of exposure to explicit sexual material on early sexual debut. ^28^

The study findings reveal that majority agreed that early sexual debut resulted from peer pressure. Our findings reenforce the fact that when teenagers associate with antisocial peers and peers who perceive norms that favor sex, they will likely initiate early sex. These findings concur with other studies where teenagers are more pressured by their age mates of the same socio-demographics to engage in sex.^29,30,31^

In this study, majority of the students never thought that they were still too young to initiate sex activities. Sexual debut was equally distributed across all age groups. It appears that the age at sexual debut will continue reducing if this attitude continues. These findings are supported by a study among Chinese women where there was a trend towards earlier sexual debut and riskier sexual behaviors in younger age groups. ^32^

Still, most students believed engaging in sex does not go against religious beliefs indicating a reduced influence of religion on sexuality among the students. In Kenya, males appear to fear pregnancy compared to females. This is contradictory considering that the girls bear the biggest burden in case of pregnancy. Despite most females, knowing that early sexual debut may result in school dropout, 71(57.7%) reported early sexual debut. This finding could be explained by the various efforts by stakeholders in Kenya to implement the world health organization guidelines aimed at preventing early pregnancy through increasing access to and use of contraception and increasing knowledge of the importance of pregnancy prevention.^33^ The fear of HIV infection appeared moderate and sexual debut was high, 71(70.3%) among those who knew sex would result in HIV infection. This could be an indication of reduction in stigma associated with HIV infection in Kenya. However, the nonchalant attitude towards the risk of HIV infection is of concern since more new HIV infections in Kenya occur among this age group. Adolescents who engage in in early sexual activities rarely use any contraceptive method of protection, ^29^. The risk of HIV infection is high considering that the reported HIV testing rates for Kenya are lowest among adolescents between 15–19 years.^3^

## CONCLUSION

Early sexual debut among adolescents was high (60.48%). The high early sexual debut was influenced by socioeconomic factors such as peer pressure, social media and poverty. Patterns of early sexual debut differs between males and females. Males experience debut with younger females while females experience with older males

### Limitations of the study

The dependent variable –early sexual debutis problematic to objectively define as one would wonder how young is too early since several girls are married at an early ag in Kenya.

This study relied on self-reports of sexual behavior which may be affected by respondent recall bias. Self-recall bias may result in lower prevalence of early sexual debut than the true prevalence.

## RECOMMENDATIONS

**To the Ministry of Health:** The high prevalence of early sexual debut calls for a policy shift in the ministry of health regarding prevention of diseases such as human papillomavirus-related cervical cancer.

**The state and non-state actors:** The socioeconomic determinants require the state and non-state actors to come up with interventions to limit social exposures that encourage early sexual debut.

**To parents:** Parent sshould be more involved in their childrens' sexual education to guide them on sexual morals.

**To researchers:** Further research may be carried out to explore the gender differences in the patterns of early sexual debut.
